# Mangiferin Alleviates Postpartum Depression–Like Behaviors by Inhibiting MAPK Signaling in Microglia

**DOI:** 10.3389/fphar.2022.840567

**Published:** 2022-06-03

**Authors:** Meichen Yan, Xuena Bo, Xinchao Zhang, Jingdan Zhang, Yajin Liao, Haiyan Zhang, Yong Cheng, Junxia Guo, Jinbo Cheng

**Affiliations:** ^1^ Center on Translational Neuroscience, College of Life and Environmental Science, Minzu University of China, Beijing, China; ^2^ Key Laboratory of Modern Preparation of TCM, Ministry of Education, Jiangxi University of Traditional Chinese Medicine, Nanchang, China; ^3^ Beijing Key Laboratory of Bioactive Substances and Functional Foods, Beijing Union University, Beijing, China; ^4^ The Brain Science Center, Beijing Institute of Basic Medical Sciences, Beijing, China

**Keywords:** postpartum depression, mangiferin, microglia, neuroinflammation, MAPK signaling

## Abstract

Postpartum depression (PPD), a severe mental health disorder, is closely associated with decreased gonadal hormone levels during the postpartum period. Mangiferin (MGF) possesses a wide range of pharmacological activities, including anti-inflammation. Growing evidence has suggested that neuroinflammation is involved in the development of depression. However, the role of MGF in the development of PPD is largely unknown. In the present study, by establishing a hormone-simulated pregnancy PPD mouse model, we found that the administration of MGF significantly alleviated PPD-like behaviors. Mechanistically, MGF treatment inhibited microglial activation and neuroinflammation. Moreover, we found that MGF treatment inhibited mitogen-activated protein kinase (MAPK) signaling *in vivo* and *in vitro*. Together, these results highlight an important role of MGF in microglial activation and thus give insights into the potential therapeutic strategy for PPD treatment.

## Introduction

Postpartum depression (PPD) is a mental health disorder that frequently occurs in women during the postpartum period. The disorder is characterized by emotional changes, including melancholic and languid mood, low self-evaluation, lack of confidence, and even suicidal tendencies. Self-harm behaviors have been reported to be common in PPD patients, ranging from 5 to 14% (Lindahl et al., 2005). The average prevalence rate of PPD was previously reported to be approximately 13% (Weissman et al., 2004); however, recent studies have shown that the global prevalence rate of PPD was higher than the earlier estimate varying across countries (Hahn-Holbrook et al., 2017). Currently, drugs for the treatment of PPD in clinics are mainly monoamine oxidase inhibitors (MAOIs), tricyclic antidepressants, and selective 5-HT reuptake inhibitors (SSRIs). However, owing to the associated side effects, such as anorexia, nausea, diarrhea, headache, nervousness, anxiety, and insomnia (Gjerdingen, 2003), the development of new anti-PPD drugs with higher efficacy and fewer side effects is urgently needed.

The levels of progesterone and estrogen increase steadily during pregnancy but decrease rapidly and remain at lower levels for a long time after childbirth (Hendrick et al., 1998). Dramatic changes in postpartum gonadal hormone levels are thought to be an important reason for the occurrence of PPD in the clinic. Based on this theory, multiple studies have established a PPD animal model by injecting progesterone and estrogen to mimic postpartum gonadal hormone changes (Zhang S et al., 2017; Zhu and Tang, 2020; Zhang et al., 2021). However, to date, the potential etiology of PPD has remained unclear, and the regulatory mechanisms are largely unknown. Growing evidence has suggested that neuroinflammation is involved in the development of depression. Increased levels of inflammatory cytokines, such as interleukin-1 beta (IL-1β), IL-8, and tumor necrosis factor-α (TNF-α), have been found in depressed patients in the clinic (Bauer et al., 2014; Walker et al., 2014). Microglia are one of the major types of immunological cells in the central nervous system and are involved in multiple neurological diseases, including Alzheimer’s (Heneka et al., 2013; Pan et al., 2019; Cheng et al., 2021), Parkinson’s (Gao et al., 2002; Lee et al., 2018; Cheng et al., 2020), and stroke (Zhao et al., 2016; Liao et al., 2020). For mental health disorders, it has been documented that microglial activation and NLRP3 inflammasome contribute to the development of post-traumatic stress disorder (Dong et al., 2020; Li S et al., 2021). In addition, it has been reported that the knockout of Dlg1 in microglia alleviated LPS-induced depression in mice by inhibiting microglial activation and neuroinflammation (Peng et al., 2021). Recently, too, neuroinflammation was reported to be involved in PPD pathology (Kendall-Tackett, 2007; Maes et al., 2000; Anderson and Maes, 2013; O'Mahony et al., 2006; Zhang X. L et al., 2017).

Mangiferin (MGF) is a type of tetrahydroxy pyrone carbonate, which can be extracted from several plants, such as *Mangifera indica L* and *Amygdalus communis L*. MGF possesses a wide range of pharmacological properties, including antitussive, anti-asthmatic, antiviral, immunoregulatory, antitumor, and anti-inflammatory activities (Saleh et al., 2014; Sellamuthu et al., 2014; Benard and Chi, 2015; Jang et al., 2016; Shi et al., 2016; Fan et al., 2017). In this study, we established a hormone-simulated pregnancy PPD mouse model and found that MGF alleviated PPD-like behaviors in mice. Mechanistically, MGF inhibited mitogen-activated protein kinase (MAPK) signaling *in vivo* and *in vitro*, thus inhibiting microglial activation and neuroinflammation.

## Results

### MGF Treatment Alleviates Depression-Like Behavior

**FIGURE 1:**
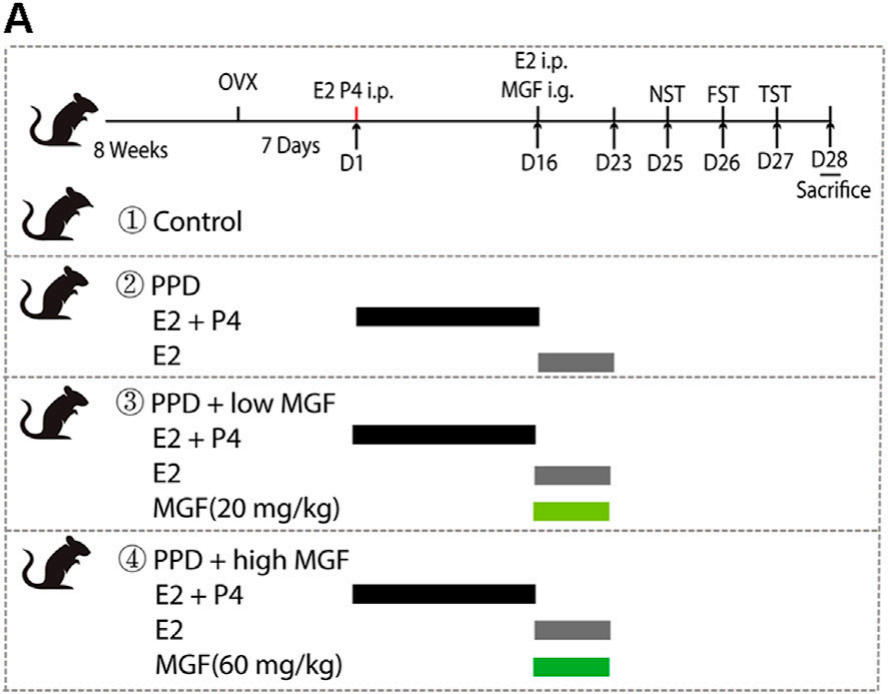
Timeline of experimental design, including the schedule of the hormone-stimulated pregnancy (HSP)-induced postpartum depression (PPD) mice model and drug administration and behavior tests. Female BALA/c mice were ovariectomized bilaterally for 7 days. The ovariectomized mice were injected intraperitoneally with β-estradiol (E2, 0.5 μg/day) and progesterone (P4, 0.8 mg/day) for 16 consecutive days. Progesterone was then withdrawn, and a high dose of β-estradiol (10 μg/day) was administrated alone. At the same time, two-dose concentrations of mangiferin (MGF) were administrated to the treatment group mice.

**FIGURE 2:**
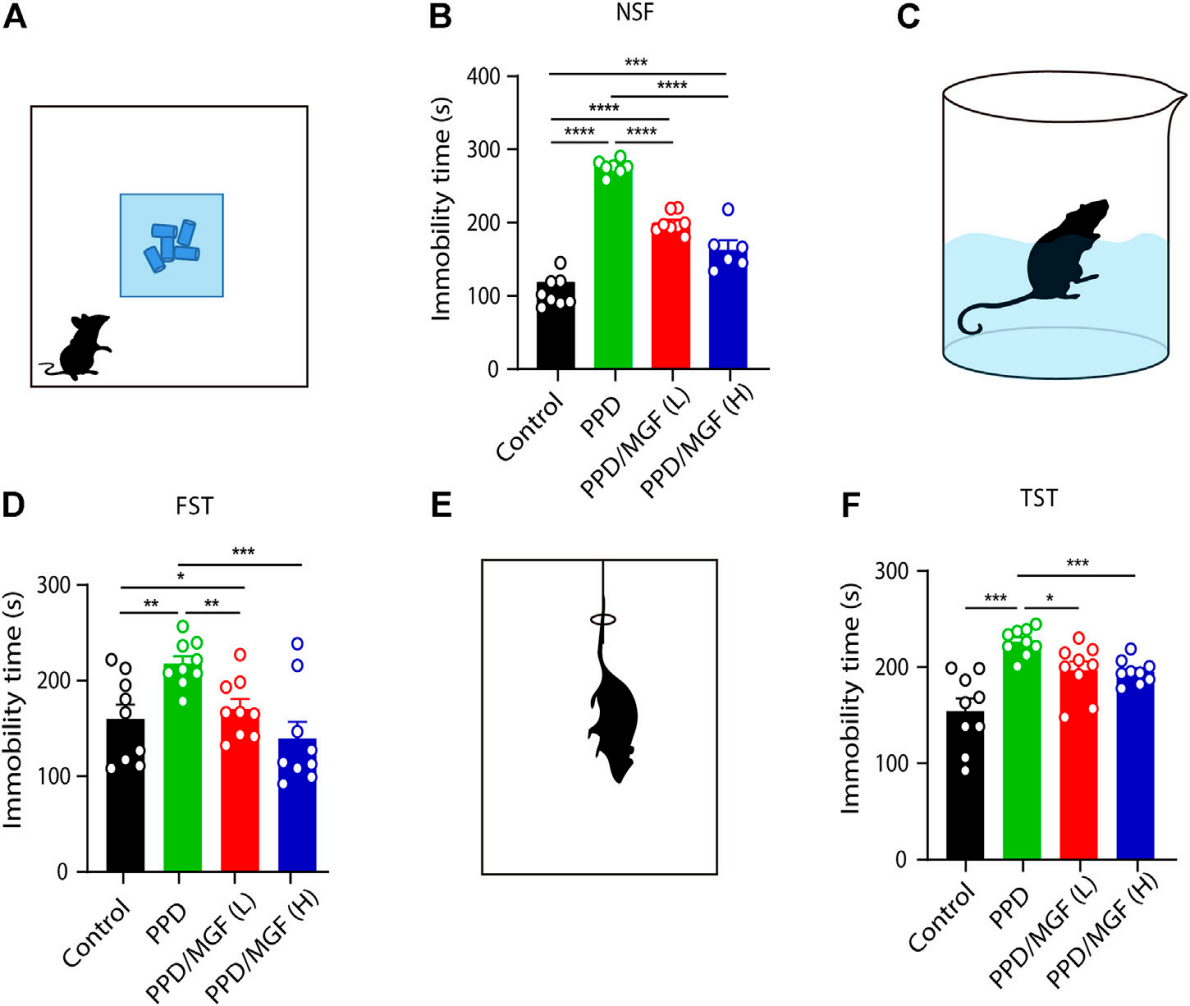
MGF-alleviated HSP-induced depression-like behavior in mice. **(A)** Schematic representation of the novelty suppressed feeding test (NST). **(B)** Analysis of immobility time in the NST [control, n = 8, PPD, n = 7, PPD/MGF (L), n = 8, and PPD/MGF (H), n = 6]. **(C)** Schematic representation of the forced swim test (FST). **(D)** Analysis of immobility time in the FST, n = 9 in each group. **(E)** Schematic representation of the tail-suspension test (TST). **(F)** Analysis of immobility time in the TST, n = 9 in each group; Error bars are mean ± S.E.M. *p < 0.05, **p < 0.01, and ***p < 0.001.

To study the effects of MGF on PPD, we established a hormone-simulated pregnancy (HSP) mouse model combined with ovariectomy (OVX). As shown in Figure 1, behavioral tests began 10 days after progesterone (P4) withdrawal. Two doses of MGF (20 and 60 mg/kg) were orally administered once per day. Moreover, the novelty-suppressed feeding (NSF) test was used to evaluate exploration and anhedonia behaviors, while the forced swim test (FST) and tail-suspension test (TST) were utilized to assess depression-like behaviors. We found that mice in the PPD model group showed increased immobility time in the NSF test, FST, and TST (Figures 2A–F), indicating impaired emotional functions. Interestingly, administration of MGF significantly decreased the immobility time in the NSF test in a dose-dependent manner compared with the PPD group (*p* < 0.001) (Figures 2A,B). Consistently, administration of MGF significantly decreased the immobility time in the FST and TST, suggesting alleviated depression-like behaviors (Figures 2C–F). Furthermore, we compared the PPD/MGF groups with the control groups through behavioral tests and found that PPD/MGF groups reduced the immobility time of PPD mice in NSF, which was still higher than that in the control groups. However, there was no difference in immobility time between high doses of the MGF and the control group in TST and FST, indicating a protective effect of MGF. Collectively, these results suggest that the administration of MGF could alleviate HSP-induced depression-like behavior in mice.

### MGF Treatment Decreases Inflammatory Cytokine Levels in the Mouse Brain

**FIGURE 3:**
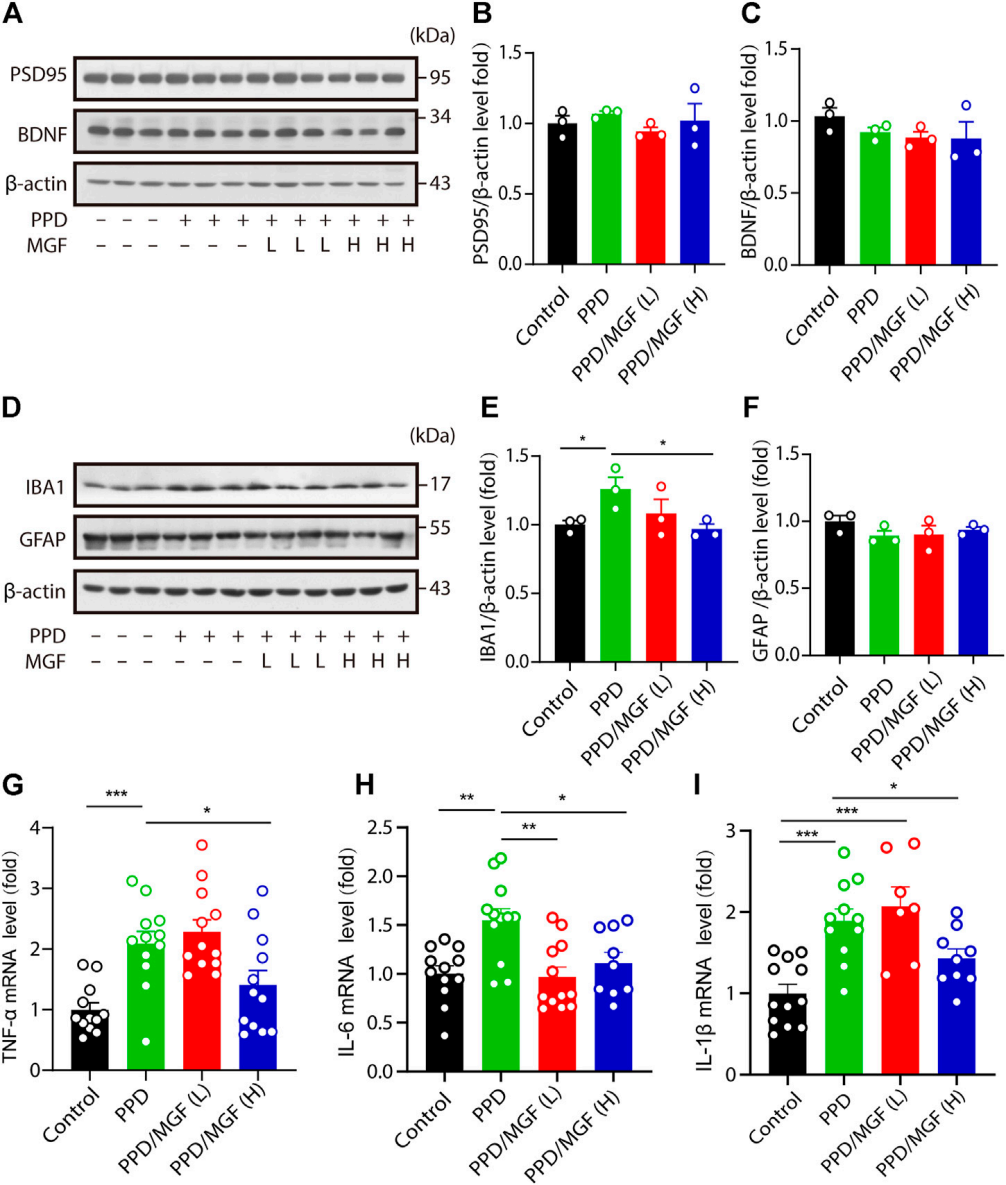
MGF decreased inflammatory cytokine levels in the mouse brain. **(A–C)** Immunoblotting and quantitative analysis of plasticity-related protein 95 (PSD95) and brain-derived neurotrophic factor (BDNF) levels in the hippocampus of mice. **(D–F)** Immunoblotting and quantitative analysis of IBA1 and GFAP protein levels in the cortex of the indicated-group mice. **(G–I)** RT-PCR analysis of TNF-α, IL-6, and IL-1β mRNA levels in the hippocampus of mice. Error bars are mean ± SEM. *p < 0.05, **p < 0.01, and ***p < 0.001.

To further study the mechanism underlying the protective effect of MGF, we examined the expression of synaptic plasticity–related protein 95 (PSD95) and brain-derived neurotrophic factor (BDNF) in the hippocampus. However, no significant differences were observed between the MGF-treated groups and the PPD model groups (Figures 3A–C). Multiple studies have suggested that neuroinflammation is involved in the development of depression (Engler et al., 2017; Moisan et al., 2021). To determine whether neuroinflammation is involved in this process, we first examined the protein levels of IBA1 and GFAP in the mouse brain. We found that the expression level of IBA1 was increased in the PPD group compared to that in the control group. MGF treatment inhibited this increase in a dose-dependent manner. There were no significant changes in the protein levels of GFAP among the four groups (Figures 3D–F). Moreover, we found that the levels of inflammatory cytokines TNF-α, IL-6, and IL-1β were significantly increased in the PPD group mice. Interestingly, treatment with MGF significantly inhibited the increase in the levels of these cytokines (Figures 3G–I). Together, these results show that MGF treatment inhibited inflammatory cytokine levels in the PPD mouse brain.

### MGF Treatment Inhibits Microglia Numbers in the Mouse Brain

**FIGURE 4:**
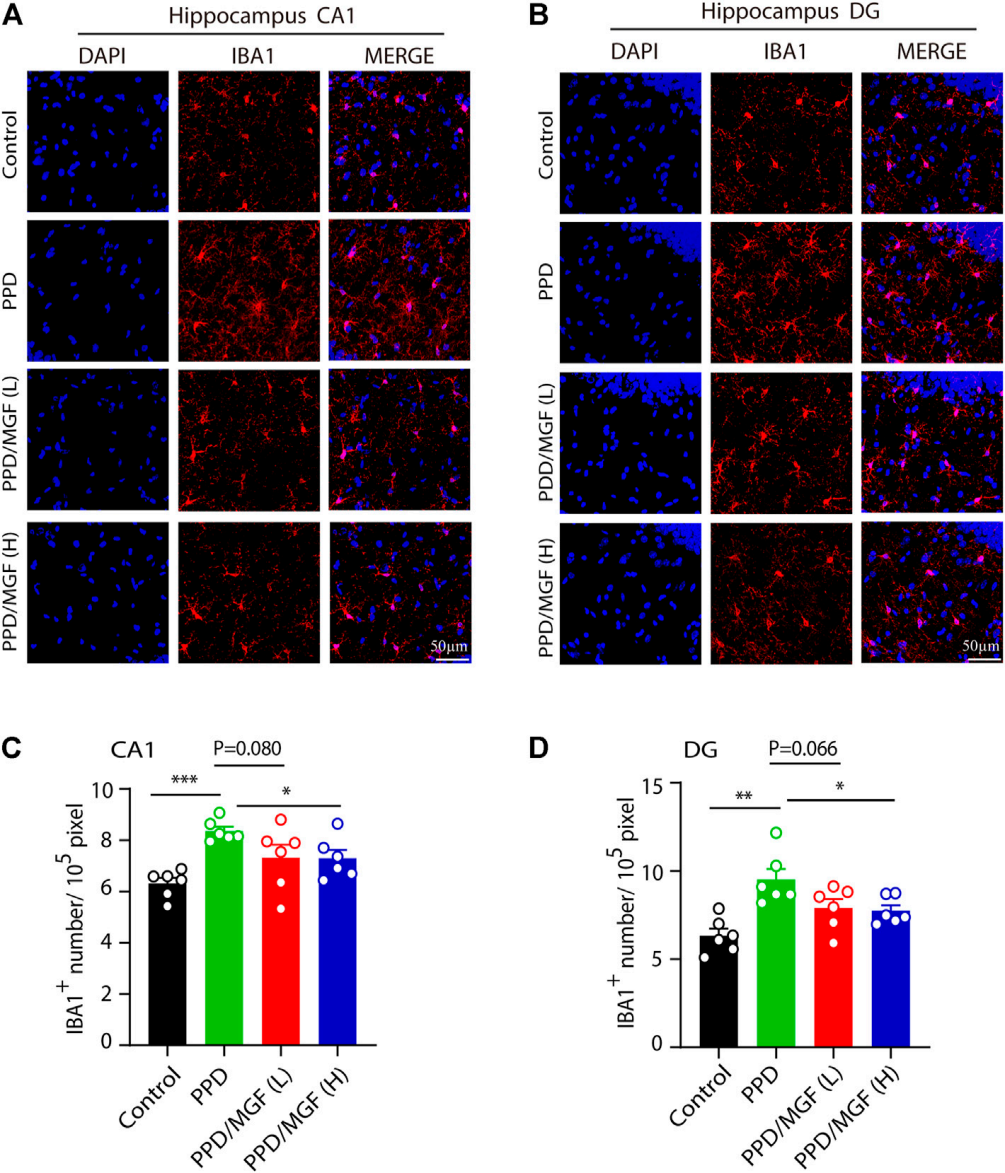
MGF inhibited microglial numbers *in vivo*. **(A–B)** Immunofluorescent staining of IBA1 in CA1 and DG areas of the hippocampus. The scale bar represents 50 μm. **(C–D)** Quantitative analysis of IBA1 cell numbers. Error bars are mean ± SEM. *p < 0.05, **p < 0.01, and ***p < 0.001.

Next, we investigated whether microglial activation is involved in this process. To address this, we performed an IBA1 immunofluorescence staining assay, which showed that a higher number of microglia existed in the hippocampus of PDD mice (Figures 4A,B). Using Image-Pro Plus software, we analyzed the number of microglia in the CA1 and DG areas of the hippocampus in these four groups of mice. The number of microglia was significantly increased in the CA1 and DG areas of the hippocampus in the PPD group mice (*p* < 0.001 and *p* < 0.01, respectively), while treatment with a high concentration of MGF significantly inhibited this increase, with a decreasing trend seen in the low concentration of MGF treatment groups (Figures 4C,D). Thus, these results suggest that microglia were activated in the PPD model mouse brain and that MGF treatment could significantly inhibit microglial activation.

### MGF Inhibits Microglial Activation by Targeting MAPK Signaling

**FIGURE 5:**
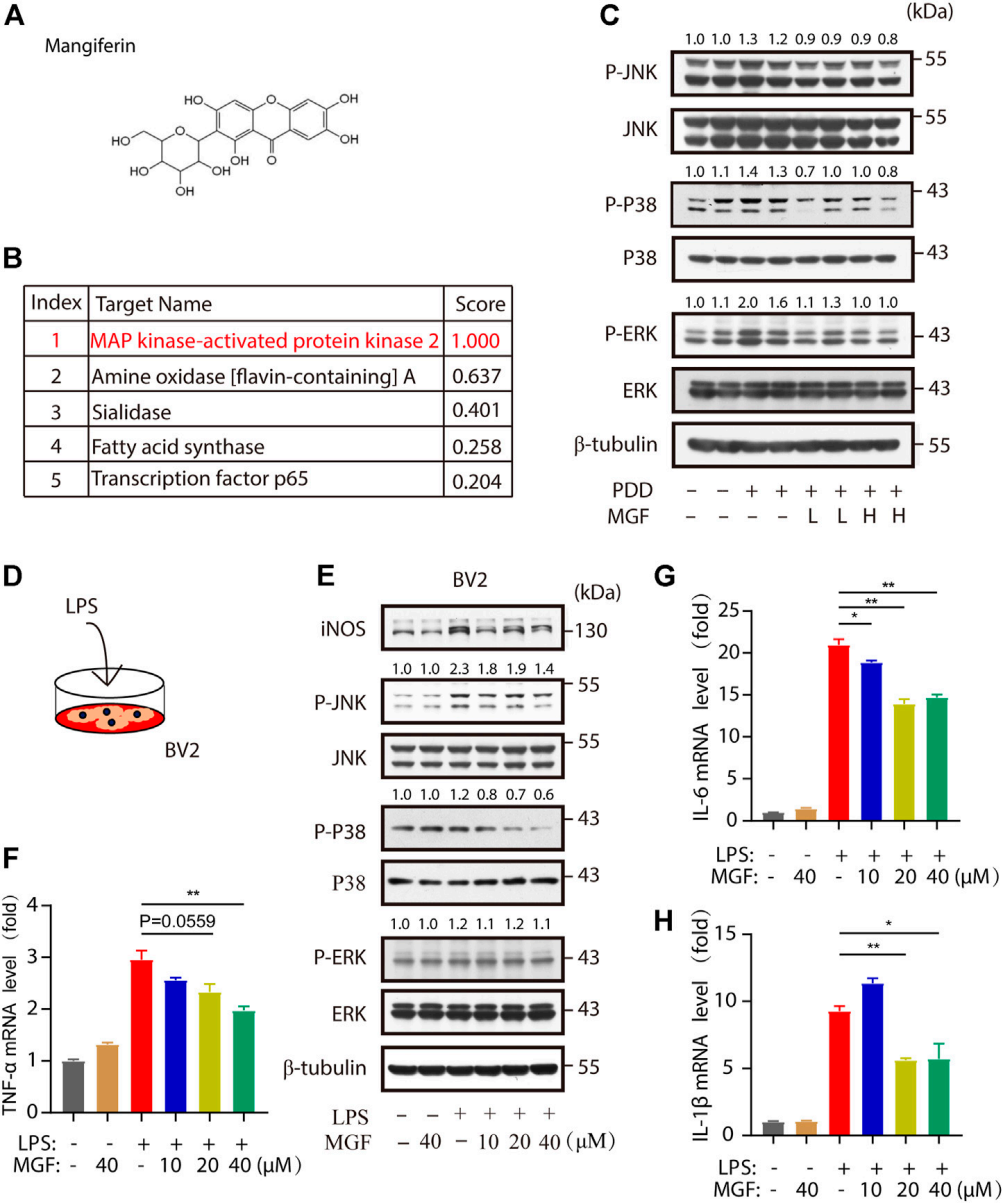
MGF-regulated mitogen-activated protein kinase (MAPK) signaling *in vivo* and *in vitro*. **(A)** MGF structure. **(B)** Potential protein targets of MGF ranked by the standard score of the probabilities. **(C)** Immunoblotting analysis of p-JNK, JNK, p-p38, p38, p-ERK, ERK, and β-tubulin protein levels in the hippocampus of mice. The number represents the normalized quantitative value of the protein. **(D)** The schematic representation of LPS stimulation in BV2 cells. **(E)** Immunoblotting analysis of iNOS, p-JNK, JNK, p-p38, p38, p-ERK, ERK, and β-tubulin protein levels from BV2 cells after being treated with MGF for 0.5 h and then stimulated LPS (1 μg/ml) for 6 h. The number represents the normalized quantitative value of the protein. **(F–H)** RT-PCR analysis of TNF-α, IL-6, and IL-1β mRNA levels in BV2 cells after being treated with MGF for 0.5 h and then stimulated LPS (1 μg/ml) for 6 h. Error bars are mean ± SEM. *p < 0.05, **p < 0.01, and ***p < 0.001.

To find the potential molecular targets of MGF, bioinformatic analysis of 3D similarity searching, ranking, and superposition was performed using ChemMapper (http://www.lilab-ecust.cn/chemmapper/index.html). Among the predicted targets (MAP kinase–activated protein kinase 2, amine oxidase [flavin-containing] A, sialidase, fatty acid synthase, and transcription factor p65), MAP kinase–activated protein kinase 2 (MAPK) was ranked first, with a 3D similarity score of 1.0 (Figures 5A,B). Next, to study changes in MAPK signaling in the hippocampus of the mouse brain, the levels of p-JNK, p-p38, and p-ERK were investigated. As shown in Figure 5C, increased levels of these three markers were observed in the PPD group compared to the control group. Notably, administration of MGF inhibited the increase in p-JNK, p-p38, and p-ERK levels, suggesting downregulation of MAPK signaling in the mouse brain. To further confirm the effects of MGF on microglia, we cultured microglial BV2 cells and studied the effect of MGF on LPS-induced MAPK signaling activation *in vitro* (Figure 5D). As shown in Figure 5E, LPS treatment increased the protein levels of iNOS, p-JNK, and p-p38, whereas pretreatment with MGF largely inhibited increased levels. Consistently, the levels of the downstream inflammatory cytokines TNF-α, IL-6, and IL-1β were significantly inhibited in the MGF treatment group (Figures 5F–H). Collectively, these results show that MGF inhibits microglia-mediated inflammation by targeting MAPK signaling.

**FIGURE 6:**
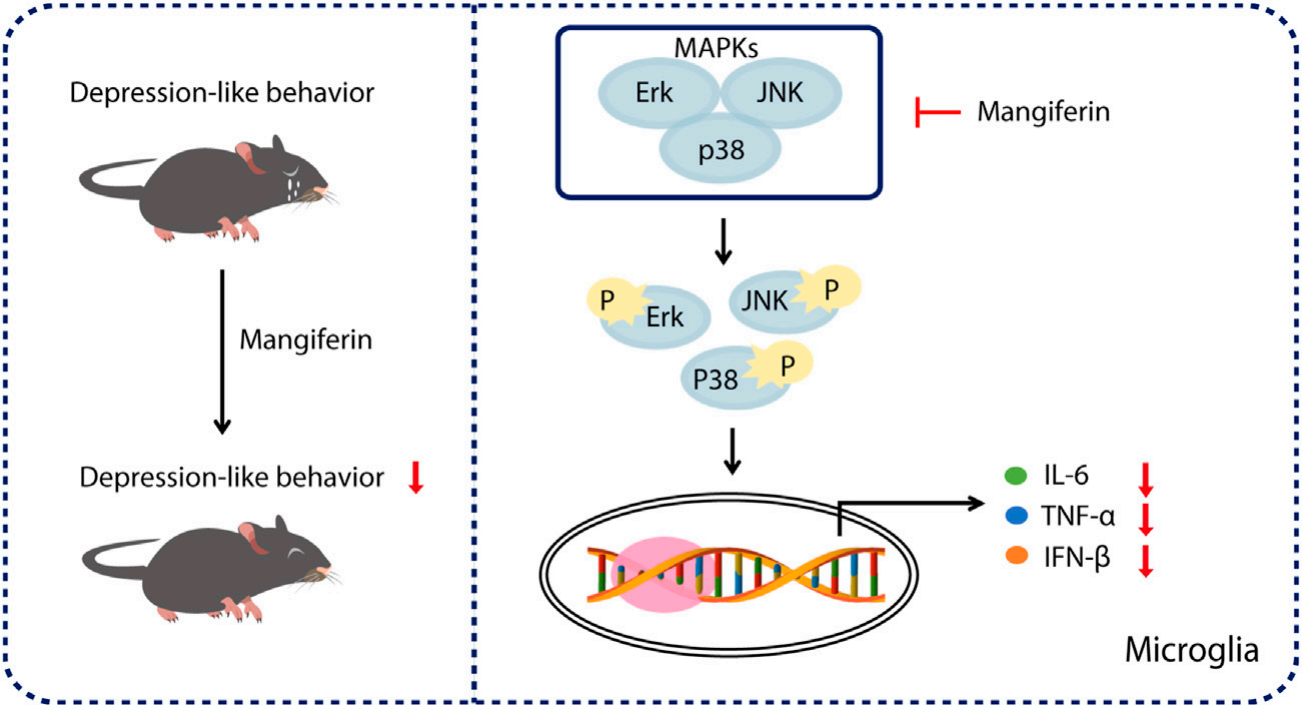
Schematic representation of the mechanism of MGF in treatment of PDD in mice. Treatment of MGF could significantly alleviate the HSP-induced PPD-like behaviors in mice. Mechanistically, MGF inhibited microglial activation by targeting MAPK signaling *in vivo* and *in vitro*.

In summary, our results show that treatment with MGF significantly alleviated PPD-like behaviors in mice. Mechanistically, we found that MGF inhibited microglial activation by targeting MAPK signaling *in vivo* and *in vitro* (Figure 6), providing a potential therapeutic strategy for PPD treatment.

## Discussion

As a common but severe mental health disorder, PPD poses a serious global burden worldwide. Multiple animal models of PPD have been established to explore its pathogenesis, including stress-induced (Boccia et al., 2007; Haim et al., 2016), HSP-induced (Stoffel and Craft, 2004; Schiller et al., 2013), and transgenic animal models (Tillmann et al., 2019; McDonnell et al., 2020). Among these, the HSP-induced model is commonly used due to its advantages such as good reproducibility and easier procedure. In this study, increased immobility times were found in the NSF test, FST, and TST in the PPD model group mice, indicating impaired emotional functions. Based on this mouse model, we found that MGF significantly alleviated PPD-like behaviors. Mechanistically, we found that MGF modulated MAPK signaling in microglia, thus inhibiting microglial activation and neuroinflammation.

Multiple studies have shown that reproductive hormone levels rapidly decline after delivery and are considered the main contributor to the occurrence of PPD (Bloch et al., 2000; Galea et al., 2001; Studd, 2015). Neuroinflammation, GABAergic inhibition, and hippocampal neurogenesis impairment are associated with the development of PPD (Zhang et al., 2016; Yang et al., 2017; Zhu and Tang, 2020). In this study, we found no significant changes in the levels of synaptic plasticity–related proteins PSD95 and BDNF in the hippocampus of PPD group mice. However, the IBA1 levels, a microglial marker, were significantly increased, and higher levels of the inflammatory cytokines TNF-α, IL-6, and IL-1β were also noted, suggesting involvement of neuroinflammation. IL-6 and IL-1β levels have been reported to be positively correlated with depression scores in postpartum women (Cassidy-Bushrow et al., 2012). Herein, the dose of MGF was determined based on previous *in vivo* experiments. Administration of 20 mg/kg of MGF possesses several beneficial biological activities, including inhibition of mastitis induced by LPS (Qu et al., 2017), ameliorating learning deficits (Jung et al., 2009), and antidepressant effects in a chronic mild stress mouse model (Cao et al., 2017). Moreover, concentrations of 30, 40, and 60 mg/kg were used in previous studies (Jangra et al., 2014; Song et al., 2020). Therefore, concentrations of 20 and 60 mg/kg MGF were chosen for this study. Notably, we found that treatment with MGF effectively suppressed the increase in inflammatory levels and alleviated HSP-induced depression-like behavior in mice, suggesting that the beneficial role of MGF in PPD may be due to its anti-inflammatory effects.

As resident immune cells of the central nervous system, microglia play a critical role in neuroinflammation. Microglial activation is closely associated with neurodegenerative diseases, strokes, and psychiatry disorders (Dong et al., 2020; Liao et al., 2020; Li S et al., 2021; Cheng et al., 2021). Here, we found that the number of microglia significantly increased in the hippocampus of the PPD group mouse brain, suggesting that microglial activation might be involved in the development of PPD. Moreover, treatment with MGF significantly inhibited the increase in microglial number in the hippocampus, suggesting that the neuroprotective role of MGF might be associated with its inhibitory effect on microglial activation. To further elucidate the potential targets of MGF, we performed bioinformatics analysis and found that MGF targets MAPK signaling, which regulates cell proliferation, stress response, inflammation, cell differentiation, and apoptosis (Li Z et al., 2021; Qin et al., 2021; Wang et al., 2021; Yang et al., 2021). More importantly, the MAPK signal pathway has been linked to several diseases, including depression (Duman et al., 2007; Wang and Mao, 2019; Humo et al., 2020). In this study, we confirmed the inhibitory effect of MGF on MAPK signaling *in vivo* and *in vitro*. Nevertheless, further regulatory mechanisms must be clarified in the future.

Our results demonstrate that treatment with MGF attenuated HSP-induced PPD-like behaviors in mice. Mechanistically, we found that MGF suppressed microglial activation by targeting and inhibiting MAPK signaling activation, thus inhibiting downstream inflammatory cytokine levels, suggesting a potential therapeutic target for the clinical treatment of PPD.

## Material and Methods

### Reagents and Antibodies

MGF (purity ≥98%) was purchased from Chengdu Desite Biotechnology (Chengdu, China). β-estradiol (E8875), dimethyl sulfoxide (DMSO), and LPS were purchased from Sigma-Aldrich (St. Louis, MO, United States). Progesterone was obtained from VETEC (V900699). The antibodies used for western blotting were as follows: Iba1/AIF-1 (E4O4W) (#17198), GFAP (E4L7M) (#80788), PSD95 (D27E11) (#3450), BDNF (#47808), iNOS (D6B6S) (#13120), anti-p-ERK1/2 (Thr202/Tyr204) (#9101), anti-ERK1/2 (#9102), anti-p-p38 MAPK (Thr180/Tyr182) (#4511), anti-p38 MAPK (#9212), and anti-p-JNK (Thr183/Tyr185) (#9251) were purchased from Cell Signaling Technology (Beverly, MA, United States). β-tubulin (#CW0098A) and β-actin (#CW0096M) were procured from CWBiotech (Beijing, China).

### Mice

Female BALA/c mice (8 weeks old, 20–25 g) were housed in the animal care facility of our institute. All animal experimental procedures were approved by the Biological and Medical Ethics Committee of Minzu University of China. All mice were maintained under conditions of a 12-h light/dark cycle at 23°C and were provided with food and water.

### Cell Culture and Treatment

BV-2 microglial cell lines were maintained in Dulbecco’s Modified Eagle’s Medium (DMEM, #11965-092, Life Technologies, Waltham, MA, United States) supplemented with 10% heat-inactivated fetal bovine serum (FBS, #04-001-1A, Biological Industries, Israel) and 1% penicillin-streptomycin solution (#03-031-1B, Biological Industries) at 37°C in a humidified atmosphere with 5% CO_2_.

### PPD Model

Two-month-old female mice were chosen, and hormone-induced pseudopregnancy (HSP)-induced PPD models were established as previously described (Li et al., 2018; Zhang et al., 2021). Mice were randomly divided into four groups (control, PPD, PPD/low MGF, and PPD/high MGF). OVX was performed under isoflurane anesthesia. After 7 days of recovery from OVX operation, mice in the PPD and PPD with MGF treatment groups were intraperitoneally injected with β-estradiol (E2, 0.5 g/day) and progesterone (P4, 0.8 mg/day) dissolved in 0.1 ml sesame oil daily for 16 days, resulting in a gradual increase in the concentration of E2 and P4 in mice to mimic the increases in hormone levels. Subsequently, mice were intraperitoneally injected with E2 (10 µg/day) alone for seven consecutive days to mimic high levels of E2 during pregnancy. Meanwhile, MGF was administered intragastrically at two different doses (20 and 60 mg/kg), as indicated in Figure 1.

### NST

The NST was performed as previously described, with minor modifications (Barbieri et al., 2021). Briefly, before the test, the mice were deprived of food but had free access to water for 24 h. Each mouse was positioned into the device with food placed on white paper in the same direction and allowed to freely explore for 5 min. The immobility time of each mouse was recorded.

### FST

One day before the test, mice were allowed to swim in water for 5 min. During the test, the mice were placed in a beaker (volume, 3 L) filled with water at 23–25°C. The total test time was 6 min, and the immobility time of the mice in the last 4 min was recorded.

### TST

Mice were placed in the test room 2 h before the test and hung on the instrument with a clip. Similar to the FST, the total experimental time was 6 min, and the immobility time of the mice in the last 4 min was recorded.

### Real-Time Quantitative and Reverse Transcription-PCR

Total RNA was isolated from the hippocampus of mice in each group using a TRIzol reagent (Invitrogen, cat#15596018), and 1 μg of RNA was used to synthesize cDNA using a one-step first-strand cDNA synthesis kit (Transgen Biotech, cat#AT341). Quantitative real-time PCR was performed using a 2 × SYBR Green PCR master mix (Transgen Biotech, cat#AQ131) and an Agilent Mx3005P RT-PCR system. The expression levels of the tested genes were normalized to those of β-actin. The primers for mouse IL-1β, TNF-α, IL-6, and β-actin were as follows:

Mouse IL-1β: Forward: 5′-TGT​AAT​GAA​AGA​CGG​CAC​ACC-3′; Reverse: 5′-TCT​TCT​TTG​GGT​ATT​GCT​TGG-3′.

Mouse TNF-α: Forward: 5′-CAG​GCG​GTG​CCT​ATG​TCT​C-3’; Reverse: 5′-CGA​TCA​CCC​CGA​AGT​TCA​GTA G-3′.

Mouse IL-6: Forward: 5′-CTA​CCA​AAC​TGG​ATA​TAA​TCA​GGA-3′; Reverse: 5′-CCA​GGT​AGC​TAT​GGT​ACT​CCA​GAA-3′.

Mouse β-actin: Forward: 5′-GGCTGTATTCCC CTCCATCG-3′; Reverse: 5′-CCA​GTT​GGT​AAC​AAT​GCC​ATG T-3′.

### Western Blotting Analysis

The concentration of the extracted protein was determined using the BCA assay. Equal amounts of protein were separated by polyacrylamide gel electrophoresis (SDS-PAGE) and incubated with the primary antibody overnight at 4°C, followed by incubation with a secondary antibody (1:5,000) for 1 h at room temperature. An ECL luminescent solution was used for detection.

### Immunofluorescent Staining

After anesthesia, the mice were perfused with normal saline, and then the whole brain was isolated and fixed with 4% paraformaldehyde for 24 h and dehydrated overnight in 30% sucrose solution. Whole brain tissue was embedded in OCT and sectioned using a freezing microtome (Leica CM3050S). Tissue sections were incubated with anti–goat IBA1 antibody (1:500, WAKO, Japan) overnight at 4°C with shaking. On the following day, tissue sections were incubated with secondary antibodies for 1 h at room temperature. Finally, images were captured using a laser scanning confocal microscope (Nikon, Tokyo, Japan).

### Statistical Analysis

All data are presented as mean ± SEM. The significance of the differences was determined by the *t*-test and one-way ANOVA using GraphPad Prism (GraphPad Software, San Diego, CA, United States). *p < 0.05, **p < 0.01, and ***p < 0.001 were considered as significant.

## Data Availability

The original contributions presented in the study are included in the article/Supplementary Material; further inquiries can be directed to the corresponding authors.
